# Development of Guar Gum Hydrogel Containing Sesamol-Loaded Nanocapsules Designed for Irritant Contact Dermatitis Treatment Induced by Croton Oil Application

**DOI:** 10.3390/pharmaceutics15010285

**Published:** 2023-01-14

**Authors:** Vinicius Costa Prado, Kauani Moenke, Bárbara Felin Osmari, Natháli Schopf Pegoraro, Sara Marchesan Oliveira, Letícia Cruz

**Affiliations:** 1 Laboratório de Tecnologia Farmacêutica, Departamento de Farmácia Industrial, Centro de Ciências da Saúde, Universidade Federal de Santa Maria, Santa Maria CEP 97105-900, RS, Brazil; 2 Laboratório de Neurotoxicidade e Psicofarmacologia, Programa de Pós-Graduação em Ciências Biológicas: Bioquímica Toxicológica, Centro de Ciências Naturais e Exatas, Universidade Federal de Santa Maria, Santa Maria CEP 97105-900, RS, Brazil

**Keywords:** skin, *Sesamum indicum*, polymeric nanoparticles, antioxidant, anti-inflammatory, HET-CAM

## Abstract

Irritant contact dermatitis is usually treated with corticosteroids, which cause expressive adverse effects. Sesamol is a phenolic compound with anti-inflammatory and antioxidant properties. This study was designed to evaluate a hydrogel containing sesamol-loaded ethylcellulose nanocapsules for the treatment of irritant contact dermatitis. The nanocapsules presented a size in the nanometric range, a negative zeta potential, a sesamol content close to the theoretical value (1 mg/mL), and a 65% encapsulation efficiency. Nanoencapsulation protected sesamol against UVC-induced degradation and increased the scavenging activity assessed by ABTS and DPPH radicals. The hydrogels were prepared by thickening the nanocapsule suspensions with guar gum (2.5%). The hydrogels maintained the nanometric size of the nanocapsules and a sesamol content of approximately 1 mg/g. The HET-CAM assay classified the hydrogels as nonirritating. The in vitro release of the hydrogel containing sesamol in the nanoencapsulated form demonstrated an initial burst effect followed by a prolonged sesamol release and a lower skin permeation in comparison with the hydrogel containing free sesamol. In addition, it exhibited the best anti-inflammatory effect in the irritant contact dermatitis model induced by croton oil, reducing ear edema and inflammatory cells infiltration, similar to dexamethasone (positive control). Therefore, the hydrogel containing sesamol in the nanoencapsulated form seemed to have a therapeutic potential in treating irritant contact dermatitis.

## 1. Introduction

Contact dermatitis is a common inflammatory skin disease affecting an estimated 15% of the adult population [1]. The two main forms are allergic contact dermatitis, caused by repeated skin exposure to contact allergens, and irritant contact dermatitis, which is caused by skin contact with irritant substances [2]. The pathophysiology of irritant contact dermatitis is characterized by the release of inflammatory mediators at the injured site, such as proinflammatory cytokines, tumor necrosis factor-alpha (TNF-α) and chemokines. In sequence, the presence of inflammatory mediators results in an increase in vascular permeability, polymorphonuclear cells infiltration and edema formation [3].

Regarding pharmacological treatment, topical corticosteroids are the most frequently used drugs. However, the potential adverse effects and patient discomfort caused by repeated application should be considered [4]. Corroborating this, dexamethasone, a synthetic corticosteroid frequently used for the treatment of irritant contact dermatitis, displays adverse effects, such as rosacea, stretch marks and immunological changes [5]. Thus, the development of innovative and more effective therapies for cutaneous disorders is required.

**Figure 1:**
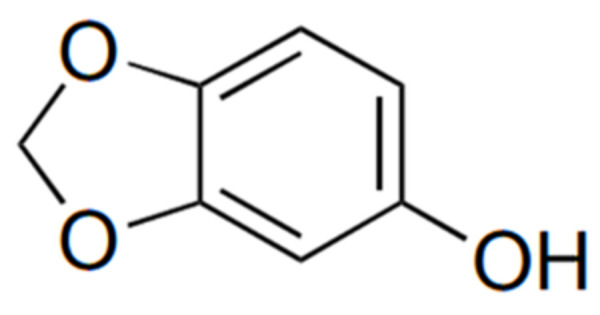
Chemical structure of sesamol.

Phenolic compounds are a resource of novel and pharmacologically active substances with a wide range of biological properties [6]. Sesamol (1,3-benzodioxol-5-ol; Figure 1), a phenolic compound extracted from roasted sesame seeds (*Sesamum indicum*), is a representative of this class that stands out because of its anti-inflammatory and antioxidant properties, as well as its low toxicological characteristic [7,8,9]. However, the conventional formulations designed for the cutaneous application of sesamol may have limitations regarding their efficacy.

Sesamol’s properties, such as small size, low molecular weight (138.2), partition coefficient of 1.29 (Log P) and water solubility (38.8 mg/mL), result in its rapid permeation through skin layers followed by systemic circulation absorption, metabolism and clearance [10]. Therefore, further studies regarding its application intending the cutaneous route are limited because of physicochemical issues and the absence of an appropriate pharmaceutical dosage form [9]. Based on these facts, the current study was designed to validate the hypothesis of developing of nano-based hydrogels using technological approaches to better explore sesamol’s therapeutic application.

Among the nanocarrier systems, polymeric nanocapsules is a nanovesicular system in which an oily core is surrounded by a polymeric shell [11]. For cutaneous treatment, polymeric nanoparticles have been used as a promising approach to circumvent physicochemical limitations and maximize the drug’ properties, allowing for prolonged drug delivery in skin layers. Since nanocapsule formulations are obtained as liquid suspensions, a gelling agent is necessary to obtain a viscosity profile suitable for cutaneous application. In this sense, natural gums have been explored due to the fact of their low toxicity and gelling and thickening properties [12]. In the present study, hydrogels containing sesamol-loaded nanocapsules were obtained using guar gum, a biocompatible and nontoxic polysaccharide [13]. Guar gum is a water-soluble polysaccharide extracted from *Cyamopsis tetragonoloba* seeds belonging to the Leguminosae family, and it widely used in the development of pharmaceutical formulations [14]. Guar gum is well recognized for its mucoadhesive properties, which increase the interaction time at the application site [15]. Accordingly, this gum was selected as the gel-forming agent in the present study. The chemical structure of guar gum is available in the Supplementary Materials (Supplementary Materials Figure S1).

Considering what was mentioned above, the present study aimed to develop a nanotechnological-based hydrogel containing sesamol and evaluate its anti-inflammatory efficacy by employing an irritant contact dermatitis model induced by croton oil in mice.

## 2. Materials and Methods

### 2.1. Reagents

Sesamol (CAS number: 533-31-1), sorbitan monooleate (Span^®^ 80), 1-1-diphenyl-2-picrylhydrazyl (DPPH) radical, 2,2′-azinobis-(3-ethylbenzothiazoline-6-sulfonic acid) (ABTS) and croton oil were obtained from Sigma Aldrich (São Paulo, SP, Brazil). The ethylcellulose polymer (Ethocel^®TM^ Standard 10 Premium Ethylcellulose) and guar gum were donated by Colorcon (Cotia, SP, Brazil) and CP Kelco (Limeira, RS, Brazil), respectively. The polysorbate 80 (Tween^®^ 80) and medium-chain triglycerides (MCTs) were provided by Delaware (Porto Alegre, RS, Brazil). Dexamethasone acetate (1 mg/g; Teuto^®^) was purchased at a commercial drugstore. Ceva (Paulínia, SP, Brazil) was the supplier of the anesthetics ketamine (Dopalen^®^) and xylazine (Anasedan^®^). Vetec (Rio de Janeiro, RJ, Brazil) was the supplier of the formaldehyde, ethanol, sodium acetate and acetic acid. Merck (Darmstadt, Germany) was the supplier of hematoxylin–eosin and paraffin. Acetone was acquired from CRQ (São Paulo, SP, Brazil). The high-performance liquid chromatography grade methanol was acquired from LiChrosolv (São Paulo, SP, Brazil). All other reagents and solvents were of analytical grade and used as received.

### 2.2. Chromatographic Method Validation for Sesamol Quantification

The quantification of sesamol was made by a HPLC-UV method in the LC-10A system (Shimadzu, Japan) composed of an LC-20AT pump, a UV-VIS SPD-M20A detector, a CBM-20A system controller and an SIL-20A HT autosampler valve. A C-18 reversed-phase column was used (Eclipse Plus, 5 μm, 110 Å, 150 × 4.60 mm (Agilent, Santa Clara, CA, USA)) with an isocratic system composed of ultrapure water and methanol (60:40) at 0.8 mL/min. The samples were injected (20 µL) into the HPLC system, and the amount of sesamol was determined at 297 nm with a retention time of 6.4 min. The assay was carried out at room temperature.

The method for the sesamol determination was validated considering the following parameters: linearity, specificity, accuracy and precision. The sesamol standard solution (1 mg/mL) was diluted at 5, 10, 15, 20 and 50 µg/mL using 40% methanolic solution (60% ultrapure water and 40% HPLC grade methanol) for the linearity evaluation (*n* = 3, using linear least squares regression analysis). The specificity of the chromatographic method was evaluated by comparison of the chromatograms obtained using a standard sesamol solution, NC SES and NC placebo. To evaluate the accuracy, a recovery assay was performed by spiking the NC placebo with the known standard sesamol solution concentrations (10, 15 and 20 μg/mL; *n* = 3). The repeatability and intermediate precision were performed in similar experimental conditions and intraday and interday analyses. Next, the relative standard deviation was calculated (RSD %; *n* = 12).

### 2.3. Preparation of the Sesamol-Loaded Polymeric Nanocapsule Suspension

The sesamol-loaded polymeric nanocapsules were produced (*n* = 3) by interfacial deposition of the preformed polymer method [16]. The organic phase, composed of ethylcellulose polymer (0.100 g), Span^®^ 80 (0.077 g), MCT oil (0.300 g) and acetone (27 mL), was kept under moderate magnetic stirring at 40 °C for 1 h. In sequence, the sesamol (0.010 g) was added to the organic phase and dissolved under stirring. After, the organic phase was injected into an aqueous phase (53 mL) containing Tween^®^ 80 (0.077 g) and maintained under magnetic stirring. After 10 min, the acetone was eliminated, and the suspension was concentrated under a reduced pressure (Rotary evaporator 558, Fisatom^®^) until a final volume of 10 mL, which corresponded to a sesamol concentration of 1 mg/mL (NC SES). For comparison purposes, formulations without sesamol (NC placebo) were also produced. After the preparation, the formulations were packaged in glass flasks and characterized in triplicate concerning the macroscopic appearance, and the granulometric distribution, average diameter, polydispersity index (PDI), zeta potential, pH, sesamol content and encapsulation efficiency (EE%) were evaluated. In addition, the stability of the developed nanocapsules suspensions was assessed by monitoring the above-cited parameters after 15 and 30 days.

### 2.4. Characterization of the Sesamol-Loaded Polymeric Nanocapsule Suspension

The granulometric distribution profile of the sesamol-loaded polymeric nanocapsules was determined by laser diffraction (Mastersizer 3000, Malvern Instruments, Bristol, UK). The polymeric nanocapsule suspension was dropped directly into the disperser compartment of the equipment containing 250 mL of distilled water until the adequate obscuration index (10–12%) was reached. The refractive index of the ethylcellulose polymer (1.47) was used for the analysis. For each nanocapsule suspension batch, the volume-weighted mean diameters (D[4;3]) were verified and are expressed in µm. In addition, the SPAN values, which represent the polydispersity of the formulations, were determined according to Equation (1):

(1) $$\mathrm{SPAN}\;=\frac{\mathrm{Dv}\;\left(90\right)-\mathrm{Dv}\;\left(50\right)}{\mathrm{Dv}\;\left(10\right)}$$

where Dv (90), Dv (10) and Dv (50) are the diameters at 90%, 10% and 50% of the cumulative distribution of the diameter curve, respectively.

The average diameter and polydispersity index (PDI) were measured using dynamic light scattering (DLS) (Zetasizer^®^ Nano-ZS ZEN 3600 model, Malvern Instruments, UK) after dilution of nanocapsules suspension (20 µL) in ultrapure water (10 mL) that was previously filtered (0.45 µm, Millipore^®^). In addition, the zeta potential was determined by electrophoretic mobility (Zetasizer^®^ Nano-ZS ZEN 3600 model, Malvern Instruments, UK). The nanocapsule suspension (20 µL) was diluted in 10 mM NaCl solution (10 mL) that was previously filtered (0.45 µm, Millipore^®^). The pH values were measured by directly immersing a calibrated electrode into the formulations (Model pH 140, Simpla, São Paulo, Brazil).

The total sesamol content in the nanocapsule suspension was determined using the HPLC method (Section 2.2). An aliquot of 150 µL sesamol-loaded polymeric nanocapsule suspension was diluted in 10 mL of 40% methanolic solution (final concentration of 15 μg/mL, corresponding to the midpoint of the standard curve). In sequence, the solution was sonicated (Ultrasonic bath Q3.0/40A model, Ultronique, Brazil) for 10 min, filtered (0.45 µm, Millipore^®^) and analyzed. The sesamol content was calculated based on a standard curve (5–25 μg/mL; r = 0.99).

To determine the encapsulation efficiency (EE%), an aliquot of 300 µL of sesamol-loaded polymeric nanocapsule suspension was placed in a 10,000 MW centrifugal device (Amicon^®^ Ultra, Millipore). The free sesamol fraction was separated by ultrafiltration/centrifugation (20 min at 2200× *g*). The ultrafiltrate was diluted 25 times with ultrapure water and analyzed using the HPLC method (Section 2.2). The difference between the total and free sesamol concentrations, determined in the sesamol-loaded nanocapsule suspension and the ultrafiltrate, respectively, was defined as EE% according to Equation (2):

(2) $$\mathrm{EE}\;\%=\frac{\mathrm{Total}\;\mathrm{sesamol}\;\mathrm{content}-\mathrm{Free}\;\mathrm{sesamol}\;\mathrm{content}}{\mathrm{Total}\;\mathrm{sesamol}\;\mathrm{content}}\times 100$$

### 2.5. In Vitro Sesamol Release Profile from the Nanocapsule Suspension

The release profile of the sesamol-loaded polymeric nanocapsule suspension was performed in triplicate using the dialysis bag diffusion method. An aliquot of the 1 mL sesamol-loaded polymeric nanocapsule suspension was placed on a dialysis bag (Sigma-Aldrich, cellulose membrane), and the system was immersed in 200 mL of 1 M potassium phosphate buffer at pH 7.4 (final concentration of 5 μg/mL) at 32 °C. At predetermined intervals (5, 15, 30, 45, 60, 120, 240, 300 and 360 min), 1 mL of the external medium was collected, and the same volume of fresh medium was replaced. The quantity of the sesamol released was assessed by the HPLC method (Section 2.2). For comparison purposes, a methanolic solution of sesamol (1 mg/mL) was also placed in the dialysis bag and simultaneously evaluated. The total sesamol content was determined based on a standard curve (0.25–10 μg/mL; r = 0.99), and the results are expressed as a % of the sesamol released.

### 2.6. In Vitro Antioxidant Potential by the Scavenging Capacity of ABTS and DPPH Radicals

The 2,2′-azinobis-(3-ethylbenzothiazoline-6-sulfonic acid) (ABTS) radical solution (0.3763 mM) was prepared by mixing 7 mM ABTS stock solution with 140 mM potassium persulfate, 24 h before the assay (final ABTS concentration of 42.7 μM). The ABTS radical concentration was 50 μM in the plate well, and ultrapure water was used as a diluent. Thus, the NC SES, free sesamol (1 mg/mL), and NC placebo samples were tested at 0.025, 0.050, 0.075, 0.10, 0.25, 0.50, 0.75, 1 and 2 μg/mL relative to the sesamol concentration.

The 1-1-diphenyl-2-picrylhydrazyl (DPPH) radical was dissolved in methanol and used as obtained (50 μM). The DPPH radical concentration was 50 μM in the plate well, and methanol was used as the diluent. Thus, the NC SES, free sesamol (1 mg/mL) and NC placebo samples were tested at 0.10, 0.25, 0.50, 0.75, 1, 1.25, 1.50, 1.75, 2 and 3 μg/mL relative to the sesamol concentration.

The samples were incubated with ABTS or DPPH solution for 30 min under light protection, and the absorbance was measured using a UV/Vis spectrophotometer (SpectraMax i3, Molecular Devices Sunnyvale, Sunnyvale, CA, USA) at 734 nm (ABTS assay) or 517 nm (DPPH assay) [16,17]. These experiments were carried out with three independent experiments in triplicate for each concentration. Moreover, an ascorbic acid aqueous solution (1 mg/mL) was used as a positive control under the same experimental conditions for the ABTS and DPPH assays. The radical scavenging activity for both assays was expressed as a % of the scavenging capacity, according to Equation (3):

(3) $$\mathrm{SC}\%=100-\frac{\left(\mathrm{Abs}\;-\mathrm{Abb}\right)}{\mathrm{Abc}}\times 100$$

where Abs is the absorbance of the incubated sample with radical, Abb is the blank sample absorbance, and Abc is the negative control absorbance.

### 2.7. Photostability Evaluation

The photostability evaluation was performed in triplicate using a mirrored chamber (1 m × 25 cm × 25 cm) coupled with an ultraviolet lamp (Phillips TUV lamp–UVC long life, 30 W; Koninklijke Philips Electronics N.V, Amsterdam, Holland), which emits ultraviolet radiation C. Aliquots of 700 µL of the free sesamol (methanolic solution of 1 mg/mL) or sesamol-loaded nanocapsule suspension (NC SES) were individually placed in plastic cuvettes with covers and exposed to UVC radiation for 1, 2, 3, 4, 5 and 6 h. For experimental validation purposes, the sesamol content in the cuvettes containing a sesamol methanolic solution of 1 mg/mL and protected from a light source with aluminum paper (dark control) were also evaluated. After the respective UVC exposure time, the remaining sesamol content was determined using the HPLC method (Section 2.2). An aliquot of 150 µL of sesamol in the free or nanoencapsulated forms was diluted in 10 mL of 40% methanolic solution (final concentration of 15 μg/mL, corresponding to the midpoint of the standard curve). In sequence, the solution was sonicated for 10 min, filtered (0.45 µm, Millipore^®^) and analyzed. The remaining sesamol content was calculated based on a standard curve (5–25 μg/mL; r = 0.99), and the results are expressed as the % of the sesamol remaining.

### 2.8. Preparation of the Hydrogels Containing Sesamol in the Free and Nanoencapsulated Forms

The hydrogels containing the sesamol-loaded nanocapsule suspensions (Hydrogel NC SES) and their respective placebo formulation (Hydrogel NC placebo) were produced in triplicate by thickening the nanocapsules suspensions with guar gum at 2.5% using a glass mortar and pestle. For the preparation of the hydrogel containing sesamol in the free form (Hydrogel free SES), the sesamol was solubilized in ultrapure water (10% *v*/*v*) and directly added to a previously 2.5% guar gum dispersion. For comparison purposes, the vehicle (Hydrogel vehicle) was prepared under the same experimental conditions, dispersing 2.5% of the guar gum with ultrapure water. Both of the hydrogels containing sesamol in the free or nanoencapsulated form presented a theoretical concentration of 1 mg/g of sesamol. After preparation, hydrogels were stored in plastic containers and characterized concerning the average diameter, polydispersity index (PDI), pH, sesamol content, spreadability profile, rheological behavior and in vitro release profile.

### 2.9. Characterization of Hydrogels

The average diameter and PDI of the nanocapsules incorporated in the hydrogels were measured by dynamic light scattering (DLS; Zetasizer^®^ Nano-ZS ZEN 3600 model, Malvern Instruments, UK). For these analyses, 0.100 g of hydrogel was kept under moderate magnetic stirring in ultrapure water (50 mL) that was previously filtered (0.45 µm, Millipore^®^) for 2 h. Next, the hydrogel dispersion was filtered through a qualitative filter and analyzed. The pH values of the hydrogels were determined by the immersion of a calibrated potentiometer (Model pH 140, Simpla, Brazil) in a dispersion of each hydrogel in ultrapure water (10% *w*/*v*). To determine the sesamol content, 0.375 g of the hydrogels was dispersed in 25 mL of 40% methanolic solution (final concentration of 15 μg/mL, corresponding to the midpoint of the standard curve). In sequence, the dispersion was sonicated (Ultrasonic bath Q3.0/40A model, Ultronique, Brazil) for 10 min and submitted to magnetic stirring for 20 min. Afterward, the dispersion was filtered (0.45 µm, Millipore^®^) and analyzed using the HPLC method (Section 2.2). The sesamol content was calculated based on a standard curve (5–25 μg/mL; r = 0.99).

### 2.10. Spreadability Profile and Rheological Behavior of the Hydrogels

The spreadability was evaluated according to the methodology described by Rigo and coworkers [18]. The aliquots of the hydrogels were placed in a central hole (1 cm in diameter) of a mold glass plate positioned on a scanner surface (HP Officejet, model 4500 Desktop). In sequence, the mold plate was carefully removed, and the circular mold was subsequently pressed with glass plates of known weights for intervals of 1 min between each plate. The spreading area images were captured at each 1 min interval employing the desktop scanner. In order to reduce bias, a single investigator performed all of the experiment. The software ImageJ (Version 1.49q, National Institutes of Health, USA) was used to calculate the captured image areas. The spreadability factor (Sf) was calculated following Equation (4):

(4) $$\mathrm{Sf}=\frac{A}{W}$$

where Sf is the spreadability factor (mm^2^/g), A is the maximum spread area (mm^2^) after the addition of the total number of plates and W is the total weight added (g).

The rheological behavior of the hydrogels was determined by employing a Brookfield viscometer (DV-I PRIME model, Brookfield, USA) using an RV07 spindle. Approximately 25 g of each hydrogel was submitted to a shear rate (s^−1^) range from 0.08, 0.10, 0.17, 0.20, 0.33, 0.50, 0.83, 1.0, to 1.67 at room temperature (25 ± 1 °C) to maintain the ideal torque (10–90%). In addition, to better elucidate the hydrogel’s rheological model, the experimental data were fitted to the following mathematical equations of Bingham (Equation (5)), Casson (Equation (6)) and Ostwald-de-Waele (Equation (7)):

(5) $$\sigma =\sigma_{0}+\;\eta \gamma$$

(6) $$\sigma^{0.5}=\sigma_{0}^{0.5}+\eta^{0.5}\gamma^{0.5}$$

(7) $$\sigma \;={\;\kappa \gamma }^{\eta }$$

where $\sigma_{0}$ is the yield stress, η is the viscosity, n is the index of flow, κ is the index of consistency, σ is the shear stress and γ is the shear rate.

### 2.11. In Vitro Sesamol Release and Ex Vivo Skin Permeation Profiles of the Hydrogels

The in vitro sesamol release and ex vivo skin permeation profiles of the hydrogels were determined using vertical Franz diffusion cells (diffusion area of 3.14 cm^2^) [19]. The receptor compartment was filled with 6 mL of the 1 M potassium phosphate buffer at pH 7.4 and kept under moderate magnetic stirring at 32 °C. After the Franz cells preparation, an aliquot of 0.5 g of the hydrogels containing free or nanoencapsulated sesamol was applied on the dialysis cellulose membrane (molecular weight of 10,000 Da; Sigma-Aldrich) or on human skin surface. The human skin sample was donated by the hospital UNIMED (Confederação Nacional das Cooperativas Médicas; Santa Maria, Brazil) resulting from the abdominal plastic surgery of one female patient. The project was approved by the committee for research with humans of the Federal University of Santa Maria (CAEE: 552200016.3.0000.5346). The subcutaneous fat was removed, and the skin was wrapped in aluminum foil and stored at −20 °C. On the day of the experiment, the skin fragments were carefully cut into circles of a similar size and fixed in the Franz diffusion cells with the stratum corneum facing upward toward the donor compartment and the dermis facing downward in contact with the receptor medium. A digital caliper was used for measuring the skin fragments thickness (3.26 ± 0.34 mm; *n* = 12).

Regarding the in vitro release, the receptor medium aliquots (0.2 mL) were withdrawn at predetermined times (5, 15, 30, 45, 60, 120, 240, 300 and 360 min), and the sesamol was quantified using the HPLC method described in Section 2.2. To maintain the experimental conditions, the same volume of fresh medium was replaced after each withdrawal. The total sesamol content was calculated based on a standard curve (0.25–50 μg/mL; r = 0.99), and the results are expressed as the sesamol released (µg/cm^2^).

For the skin permeation evaluation, the excess of hydrogel was removed after 6 h of incubation. The skin fragments and receptor media were collected, and the skin was dissected into layers for the sesamol quantification in the individual layers. The stratum corneum was removed using the tape stripping technique (18 rounds of tape; Scotch 3M^®^). In sequence, the epidermis layer was separated from the dermis by heating the skin fragment in a water bath at 60 °C for 45 s [20,21,22]. The sesamol was extracted from the skin layers with a 40% methanolic solution (4 mL for the stratum corneum, 1 mL for the epidermis and 2 mL for the dermis) followed by vortex mixing for 2 min and sonication (Ultrasonic bath Q3.0/40A model, Ultronique, Brazil) for 15 min. The samples were filtered (0.45 µm, Millipore^®^) and analyzed using the HPLC method (Section 2.2). The total sesamol content in the compartments were calculated based on a standard curve (0.25–15 μg/mL; r = 1), and the results are expressed as the µg of sesamol/cm^2^.

### 2.12. Irritant Potential Evaluation

To evaluate the irritant potential of the formulations, we used the Hen’s Egg Test (HET-CAM) [23] (*n* = 6/formulation). The experimental methodology was approved by the Ethics Committee on the Use of Animals of the Federal University of Santa Maria (CEUA; protocol number: 5428271020/2021). For the assay, fertilized chicken eggs with 10 days of incubation (37 °C and 65% relative humidity) were donated by Languiru (Teutônia, RS, Brazil). After this period, the most external shell and the white membrane were removed, and the samples were applied (300 µL for the nanocapsules suspension and 0.300 g for the developed hydrogels) on the chorioallantoic membrane (CAM). Considering the opacity of the nanocapsule suspension, a physiological solution was applied on the CAM after 20 s. In sequence, the CAM was monitored for 300 s. During this period, any modification in the membrane was monitored (vasoconstriction, hemorrhage and coagulation), and the time required for such phenomena to occur was recorded. The formulations containing sesamol in the nanoencapsulated form (sesamol-loaded nanocapsules suspension—NC SES and Hydrogel NC SES) were compared to the free sesamol form (aqueous solution of 1 mg/mL and Hydrogel free SES). To verify whether the formulation’s constituents caused any irritating effect, we tested formulations without sesamol (NC placebo, Hydrogel NC placebo and Hydrogel vehicle). For experimental validation purposes, the positive (0.1 N sodium hydroxide—NaOH) and negative (saline solution) controls were evaluated. The irritation score (IS) was determined according to Equation (8). From the IS values obtained, the formulations were classified as nonirritant (0–0.9), slightly (1–4.9), moderate (5–8.9) and severe irritant (9–21).

(8) $$\mathrm{IS}\;=\frac{\left(301-\mathrm{TIME}\;H\right)}{300}\times 5+\frac{\left(301-\mathrm{TIME}\;V\right)}{300}\times 7+\frac{\left(301-\mathrm{TIME}\;C\right)}{300}\times 9$$

where H is the hemorrhage time, V is the vasoconstriction time and C is the coagulation time.

### 2.13. In Vivo Evaluation

#### 2.13.1. Experimental Model

All experiments used male *Swiss* mice (25–30 g; 4–5 weeks of age) produced and provided by the Federal University of Santa Maria. The animals were maintained on a 12 h light–dark cycle and under a controlled temperature (22 ± 2 °C), and they were fed with standard laboratory food and water ad libitum. Before performing the experiments (8:00 a.m. and 5:00 p.m.), the animals were acclimatized to the experimental room. All experimental protocols were performed in accordance with national legislation (Guidelines of the Brazilian Council of Animal Experimentation—CONCEA) and the Animal Research: Reporting In Vivo Experiments ARRIVE guidelines (McGrath and Lilley, 2015). The experimental methodologies were approved by the Institutional Animal Care and Use Committee of the Federal University of Santa Maria (protocol number: 2665290622/2022). Herein, two different experimental sets were performed. For the inflammatory marker evaluations, 49 mice were used (7 groups/7 animals per group = 49 mice). Another 49 mice were used for the oxidative status evaluations. Therefore, 98 mice were used.

#### 2.13.2. Acute topical application of croton-oil-induced irritant contact dermatitis

The mice were separated into seven groups (7 mice per group) as described below:

(i). Naive: Mice neither received croton oil nor any treatment;
(ii). Croton oil: Mice received croton oil and no treatment;
(iii). Hydrogel vehicle: Mice received croton oil and were treated with hydrogel vehicle;
(iv). Hydrogel NC placebo: Mice received croton oil and were treated with hydrogel NC placebo;
(v). Hydrogel free SES: Mice received croton oil and were treated with hydrogel free SES;
(vi). Hydrogel NC SES: Mice received croton oil and were treated with hydrogel NC SES;
(vii). 0.5% Dexamethasone acetate: Mice received croton oil and were treated with dexamethasone acetate.

In sequence, the mice were anesthetized with a ketamine (90 mg/kg) plus xylazine (10 mg/kg) solution. Acute ear edema was induced in the mice’s right ear by a croton oil single topical application (1 mg/ear dissolved in acetone; 20 μL/ear applied with a micropipette) [24,25]. After the croton oil application, the mice’s ears were topically treated with 15 mg of hydrogels or dexamethasone (0.5%; used as a positive control). The quantity of hydrogel used in the treatment corresponded to a sesamol concentration of 1.5 µg/ear.

#### 2.13.3. Assessment of the Inflammatory Markers

The measurement of the ear thickness was performed to verify the mice ear edema by comparing the basal thickness measure with the ear measure at 6 h after the croton oil application and treatments. An increase in the ear thickness after croton oil application compared to the basal thickness indicated the occurrence of edema. A digital micrometer (Digimess, São Paulo, Brazil) was positioned in the ear of the anaesthetized animals, as previously described [24,26], to evaluate the mice’s ear thickness, which is expressed in µm. In order to reduce bias, a single investigator performed all of the measurements. After the ear thickness measurement, the mice were euthanized with thiopental (100 mg/kg, intraperitoneally), and ear biopsies were collected for further analysis.

We also evaluated the inflammatory cell infiltration of the mice’s ear tissue using a histological technique. After collection, the ears were fixed in Alfac solution (ethanol (80%), formaldehyde (40%) and acetic acid, in a proportion of 16:2:1). The ear tissue samples were embedded in paraffin, sectioned at 5 µm and stained with hematoxylin–eosin. The number of inflammatory cells infiltrated into the mice’s ear tissue was verified using a 20× objectives lens (cell number per field) and quantified using the ImageJ software [27,28].

#### 2.13.4. Assessment of the Oxidative status Markers

The samples of ears (*n* = 7/group) were homogenized (1:5, *w*/*v*) in 50 mM Tris-HCl at pH 7.4. The homogenates were then centrifuged at 3000× *g* for 10 min at 4 °C, and the supernatant was used to determine the reactive oxygen species (ROS), thiobarbituric acid reactive substances (TBARS) and nonprotein thiol (NPSH) levels.

The ROS levels were determined by the capacity to oxidize the nonfluorescent dye 2,7-dichlorofluorescein diacetate (DCFDA) to dichlorofluorescein (DCF) [29]. The supernatant samples (50 µL) were incubated in the dark, with 250 µL of 10 mM Tris-HCl buffer at pH 7.4 and 20 µL of 1 mM DCFDA for 30 min at 37 °C. The fluorescence at the excitation (488 nm) and emission (525 nm) was measured by a spectrofluorometer (SpectraMax i3, Molecular Devices Sunnyvale, CA, USA). The ROS levels were calculated using a standard curve of DCF (0.01–100 µM; r = 0.97) to determine the results, expressed as the nmol DCF/mL of the sample.

The TBARS assay was performed to indirectly determine the malondialdehyde (MDA) levels, a lipid peroxidation marker, as described by Ohkawa [30]. The MDA reacts with 2-thiobarbituric acid (TBA) under acidic conditions and high temperatures to yield chromogen. The supernatant (40 µL) was incubated with 0.8% (100 µL) acetic acid buffer (100 µL) at pH 3.4 and 8.1% sodium dodecyl sulfate (SDS) (40 µL) for 2h at 95 °C. The TBARS levels were calculated as the nmol TBARS/mL of sample, based on a standard curve of malondialdehyde (0.1–100 μM; r = 0.99).

The NPSH content, a nonenzymatic antioxidant defense, was determined using Ellman’s method [31]. Firstly, an aliquot of the supernatant was mixed (1:1) with a 10% trichloroacetic solution. The homogenates were then centrifuged at 3000× *g* for 10 min at 4 °C. After, 30 µL of supernatant containing free SH groups was added in 255 µL of 1 M potassium phosphate buffer at pH 7.4 and 15 µL of 10 mM DTNB (5,5’-dithiobis-2-nitrobenzoic acid). The color reaction was measured at 412 nm, and the NPSH levels were calculated as the nmol of NPSH/g of tissue, based on a glutathione standard curve (0.1–100 μM; r = 0.99).

### 2.14. Data Presentation and Statistical Analysis

The results are expressed as the mean ± standard deviation (SD) or the standard error of the mean (SEM). The data normality was evaluated using the Pearson omnibus normality test. The GraphPad Prism^®^ version 6 software was used to perform the unpaired student’s *t*-test or analysis of variance (ANOVA) of ordinary or repeated measures followed by the post hoc test of Tukey, when appropriate. The values of *p* < 0.05 were considered statistically significant. For the results of the ear edema (i.e., in vivo evaluations), the inhibitory effect (I) was calculated based on the response of the control group.

## 3. Result

### 3.1. Analytical Method Development

Figure S2A (Supplementary Materials File—Figure S2A) shows the sesamol chromatographic profile at 297 nm. The methodology was linear (r = 0.99 ± 0.00), accurate (101.2 ± 0.5%) and precise (relative standard deviation ≤ 1.21%) in a concentration range of 5.0–25.0 μg/mL. According to the representative chromatogram of the NC placebo, which is available in the Supplementary Materials (Supplementary Materials File—Figure S2B), the method can be considered specific, because it showed no interference from the nanocapsule suspension’s constituents in the verified sesamol peak (6.4 min). In addition, the sesamol purity peak was confirmed with a photodiode array (peak purity index > 0.99).

### 3.2. Characterization of the Sesamol-Loaded Polymeric Nanocapsule Suspension

The nanocapsule suspension showed an opalescent white color and a bluish reflection because of the Tyndall effect. The characterization parameter results are shown in Table 1. The volume-weighted mean diameters (D[4;3]) determined by the laser diffraction were lower than 0.140 µm, with SPAN values lower than two. The granulometric profile analysis showed unimodal size distribution curves with diameters especially observed in the nanometric range at their initial time. The representative images of the granulometric profile of the NC SES and NC placebo are available in the Supplementary Materials (Supplementary Materials File—Figure S3).

In sequence, the formulations were analyzed by DLS to characterize their nanometric populations. The nanocapsules showed an average diameter of less than 140 nm, and the PDI values were lower than 0.2 in accordance with the DLS analyses. The representative images of the size distribution curves determined by DLS are available in the Supplementary Materials (Supplementary Materials File—Figure S4). In addition, the nanocapsules presented negative zeta potential values and pH values in the acid range. The statistical analysis performed using the unpaired Student’s *t*-test showed no significant difference between the NC SES and NC placebo at their initial time (*p* > 0.05). The sesamol content in the formulations was close to the theoretical value (1 mg/mL). Regarding the encapsulation efficiency, the nanocapsule suspension showed 65% of the sesamol was associated with nanostructures.

After 15 and 30 days of storage at room temperature, no statistical difference was found in any physicochemical parameter tested (average diameter, PDI, PZ, pH, and sesamol content) when compared to the initial time (Table 1; *p* > 0.05, one-way ANOVA of repeated measures). However, the D[4;3] and SPAN values showed an increase for both formulations at 30 days in comparison to the initial time (Table 1; * *p* < 0.05; one-way ANOVA of repeated measures followed by Tukey’s test).

**Figure 2:**
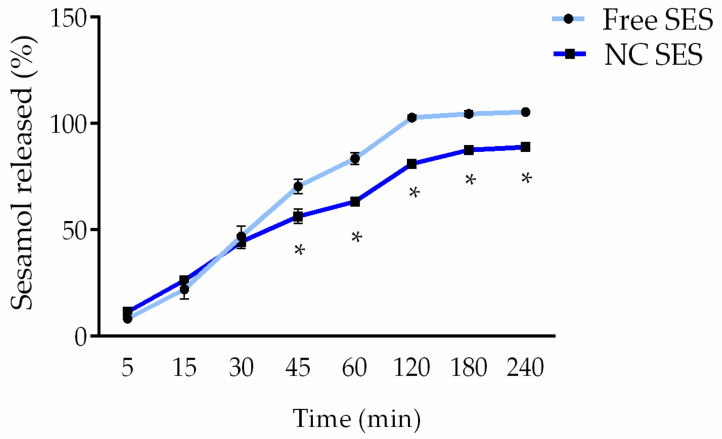
In vitro release profile of sesamol in the free (Free SES: light-blue line) or nanoencapsulated forms (NC SES: blue line). The values are reported as the mean ± SD of three independent experiments/group. All data were analyzed using the unpaired Student’s *t*-test at each point (individual time). The asterisks (*) denote a significant difference between the NC SES and Free SES (* *p* < 0.05).

Figure 2 shows the release profiles of the sesamol from the nanocapsule suspension and from its solution (i.e., free form). Until 30 min, there was no difference between both profiles. However, from 45 min, the nanocapsules demonstrated a more controlled release of sesamol (*p* < 0.05). It is possible to notice a free sesamol release of approximately 100% at 2 h, while the sesamol release from the nanocapsule suspension was approximately 81% at the same time.

### 3.3. DPPH and ABTS Scavenger Activity Evaluation

**Figure 3:**
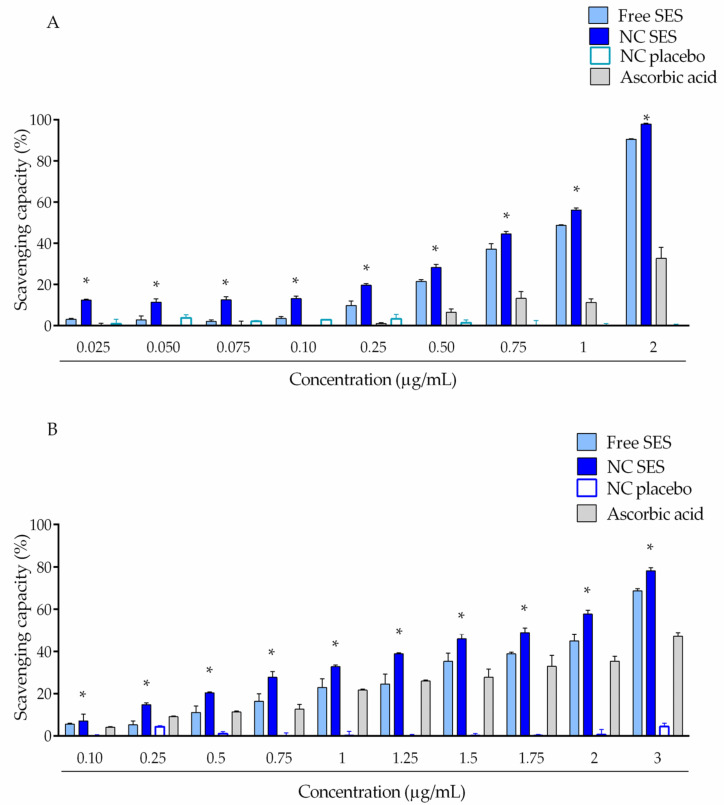
Scavenging of ABTS (**A**) and DPPH (**B**) radicals. The values are reported as the mean ± SD of three independent experiments/group. All data were analyzed by ordinary one-way ANOVA followed by post hoc Tukey’s test at each point (individual concentrations). The asterisks (*) denote the significant difference between the NC SES and Free SES (* *p* < 0.05).

The sesamol antioxidant evaluation showed ABTS (Figure 3A) and DPPH (Figure 3B) scavenging capacities higher for sesamol in its nanoencapsulated form for all tested concentrations in comparison to the free form (* *p* < 0.05; ordinary one-way ANOVA).

### 3.4. Photostability Evaluation

**Figure 4:**
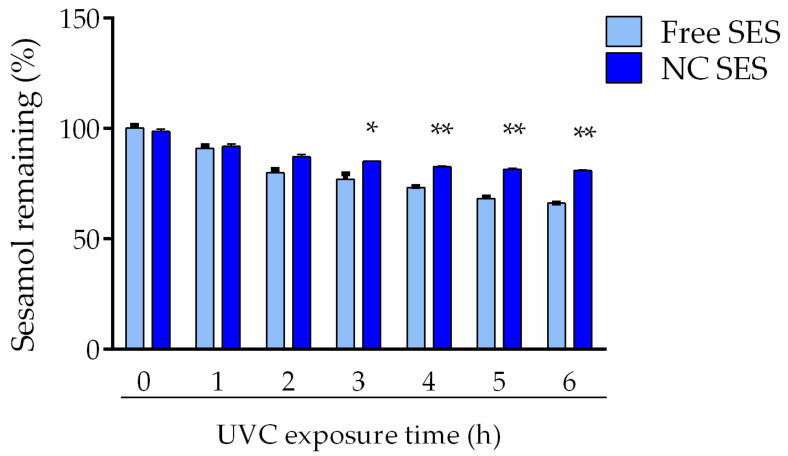
Remaining sesamol content after exposure to UVC radiation. The values are reported as the mean ± SD of three independent experiments. All data were analyzed using the unpaired Student’s *t*-test at each point (individual time). The asterisks (*) denote a significant difference between the NC SES and Free SES (* *p* < 0.05; ** *p* < 0.01).

Figure 4 displays the results of the photostability evaluation of the sesamol in the free or nanoencapsulated forms. After 360 min of UVC exposure, the sesamol content reduced to 66.1 ± 1.8% in the methanolic solution (Free SES), while the nanocapsule suspension (NC SES) had approximately 80% sesamol content, significantly higher than the methanolic solution (*p* < 0.01). No statistically significant changes in the remaining sesamol concentrations in the dark control cuvettes were observed over the experiment time.

### 3.5. Characterization of the Hydrogels

**Figure 5:**
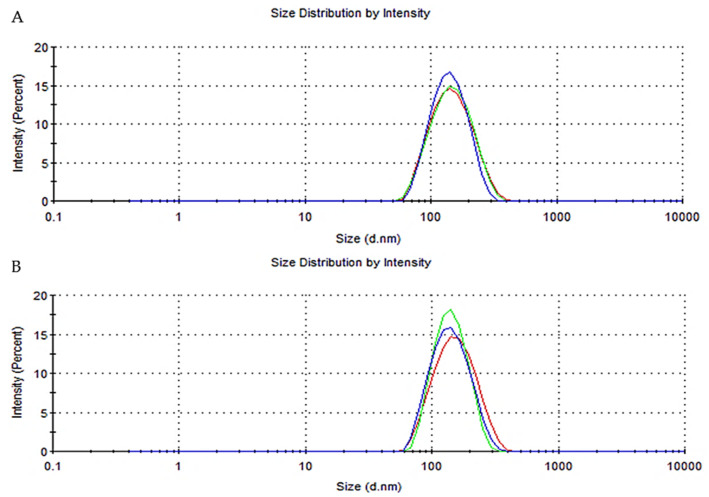
Representative images of the size distribution curves determined by dynamic light scattering (DLS) of the nanocapsules present in the Hydrogel NC SES (**A**) and Hydrogel NC placebo (**B**).

The hydrogels’ characteristics are depicted in Table 2. According to the average diameter results, the nanocapsules present in the hydrogels displayed a nanometric size similar to the original nanocapsule suspension and low PDI values. The representative images of the size distribution curves of the nanocapsules present in the hydrogels are available in Figure 5. The pH values of the hydrogels were approximately in the neutral range, and the sesamol contents were close to 99% for both hydrogels.

### 3.6. Spreadability Profile and Rheological Behavior of the Hydrogels

**Figure 6:**
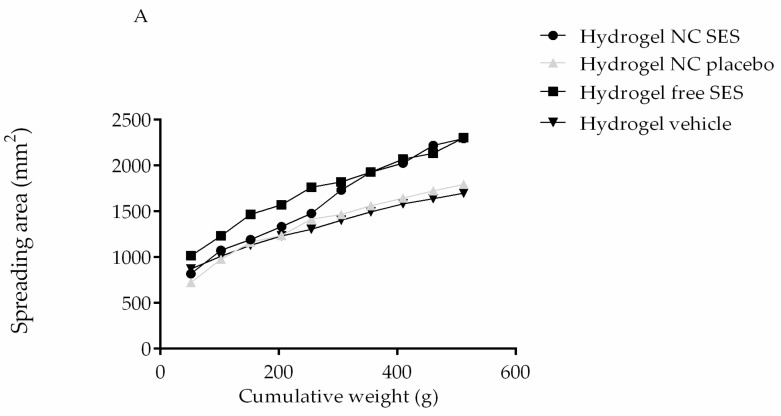

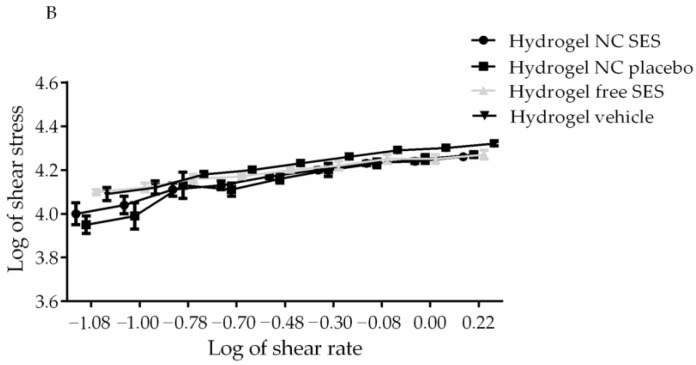
Spreadability (**A**) and Ostwald-de-Waele model (**B**) profiles of the hydrogels. The experiment was performed in triplicate for each hydrogel, and the values are reported as the mean ± SD. All data were analyzed by ordinary one-way ANOVA at each point (individual shear rate) (*p* > 0.05).

Concerning the hydrogels’ spreadability profile and rheological behavior, the results showed that the spreadability area of all of the hydrogels increased with the weight accumulation (Figure 6A). The statistical analysis showed no significant difference between the spreadability factor (Sf) of the hydrogels (Hydrogel NC SES = 4.5 ± 0.4; Hydrogel NC placebo = 3.3 ± 0.0; Hydrogel free SES = 4.50 ± 0.6; Hydrogel vehicle = 4.8 ± 0.0; *p* > 0.05; one-way ANOVA).

The rheological behavior Indicated a non-newtonian flow, with a decrease in the viscosity under shear strain. The rheograms were mathematically modeled using different equations to establish the best rheological behavior (plastic or pseudoplastic). According to the regression coefficients (Table 3), both hydrogels were better fitted to the Ostwald-de-Waele model (Figure 6B), indicating a pseudoplastic behavior. In addition, the flow index (ƞ) and consistency index (k) showed no significant difference between the hydrogels (Table 3).

### 3.7. In Vitro Release and Ex Vivo Skin Permeation Profiles

**Figure 7:**
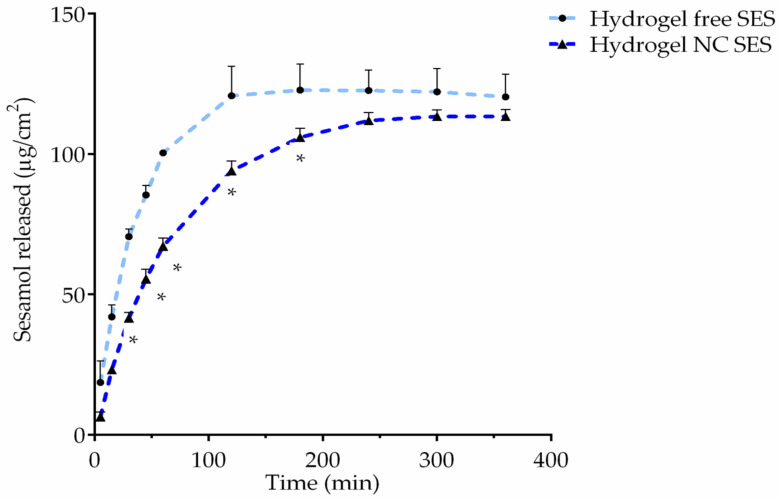
In vitro release profile of the hydrogels containing sesamol in the free (Hydrogel free SES: light-blue line) or nanoencapsulated forms (Hydrogel NC SES: blue line). The experiment was performed in triplicate for each hydrogel, and the values are reported as the mean ± SD. All data were analyzed using the unpaired Student’s *t*-test at each point (individual time). The asterisks (*) denote a significant difference between the Hydrogel NC SES and Hydrogel free SES (* *p* < 0.05).

Figure 7 shows the release profiles of the hydrogels containing sesamol in the free (Hydrogel free SES) or nanoencapsulated form (Hydrogel NC SES). The results demonstrated that the Hydrogel NC SES prolonged the sesamol release from 45 min (*p* < 0.05). In 2 h, the hydrogel released approximately 120 µg/mL of free sesamol, while 94 µg/mL of sesamol was released from the hydrogel containing nanocapsules at the same time.

**Figure 8:**
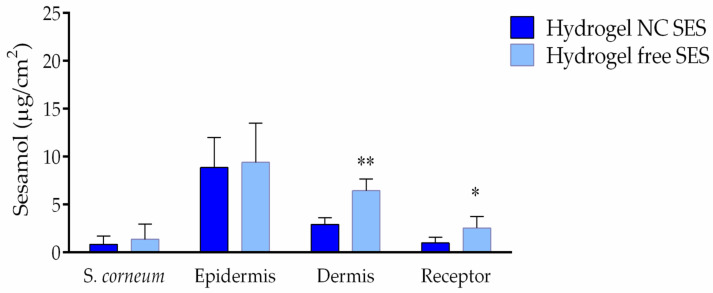
Ex vivo permeation profile of the hydrogels containing sesamol in the free (Hydrogel free SES: light-blue column) or nanoencapsulated forms (Hydrogel NC SES: blue column) after 6 h of incubation. The values are reported as the mean ± SD (*n* = 6/hydrogel). All data were analyzed by the Student’s *t*-test at each point (individual compartment). The asterisks (*) denote a significant difference between the Hydrogel NC SES and Hydrogel free SES (* *p* < 0.05; ** *p* < 0.01). *S. corneum*: *Stratum corneum* layer.

Figure 8 shows the permeation profile of the hydrogels containing sesamol in the free and nanoencapsulated forms. The sesamol content was similar in the stratum corneum layers for both hydrogels (1.37 and 0.85 μg/cm^2^ for the Hydrogel NC SES and Hydrogel free SES, respectively). The highest quantity of sesamol was detected in the epidermis layer, which presented 9.39 μg/cm^2^ for the Hydrogel free SES and 8.87 μg/cm^2^ for the Hydrogel NC SES. In the dermis layer, superior quantities of sesamol were determined in the skin samples that received the Hydrogel free SES (6.45 μg/cm^2^), while 2.93 μg/cm^2^ was quantified for the Hydrogel NC SES (** *p* < 0.01). Moreover, the formulation of the hydrogel containing free sesamol achieved a higher sesamol concentration in the receptor medium (2.54 μg/cm^2^) compared to the Hydrogel NC SES (0.98 μg/cm^2^) (* *p* < 0.05).

### 3.8. Evaluation of the Irritant Potential

**Figure 9:**
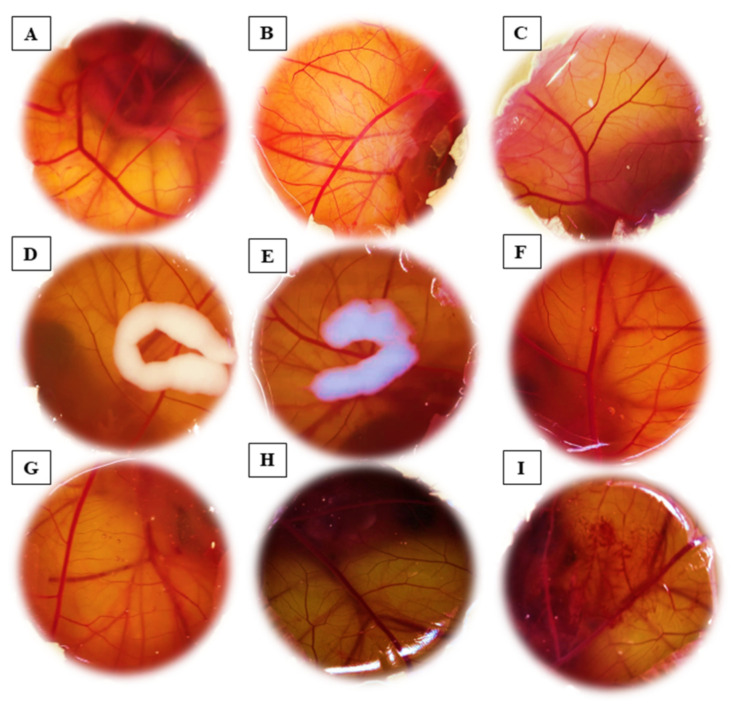
Representative images of the chorioallantoic membrane (CAM) test after the application of the (**A**) sesamol solution; (**B**) NC SES; (**C**) NC placebo; (**D**) Hydrogel NC SES; (**E**) Hydrogel NC placebo; (**F**) Hydrogel free SES; (**G**) Hydrogel vehicle; (**H**) negative control—NaCl 0.9%; (**I**) positive control—0.1 M NaOH.

Coagulation, lysis, and hemorrhage were not detected in the CAM after the application of all tested groups (IS = 0). As expected, the positive control was classified as severe irritants (IS = 20.1 ± 0.0). Figure 9 shows representative images of the CAM after the assay.

### 3.9. In Vivo Evaluations

#### 3.9.1. Antiedematogenic Effect

**Figure 10:**
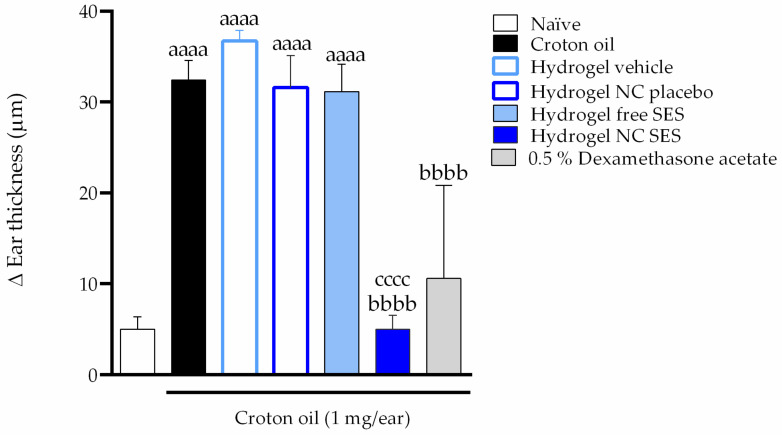
In vivo antiedematogenic effect of the hydrogels containing sesamol topically applied (15 mg/ear) in the croton-oil-induced ear edema model in *Swiss* mice. Immediately after the croton oil application, the mice’s ear received the treatments. Six hours after croton oil or croton oil plus treatments, the mice’s ear thickness was verified. Each bar represents the mean ± SEM (*n* = 7); ^aaaa^ *p* < 0.0001 shows a significant difference when compared to the naïve group; ^bbbb^ *p* < 0.0001 shows a significant difference when compared to the croton oil control group; ^cccc^ *p* < 0.0001 shows a significant difference when compared to the Hydrogel free SES group. One-way ANOVA followed by post hoc Tukey’s test.

Figure 10 shows the antiedematogenic effect of the hydrogels in the ear edema model induced by the croton oil topical application. The croton oil application increased the mice’s ear thickness to 32 ± 2 μm. The Hydrogel NC SES but not the Hydrogel free SES completely inhibited the croton-oil-caused ear thickness increase (inhibition = 100%), while 0.5% dexamethasone acetate caused an inhibition of 79.69 ± 14.09%.

#### 3.9.2. Assessment of the Inflammatory Cells’ Infiltration

**Figure 11:**
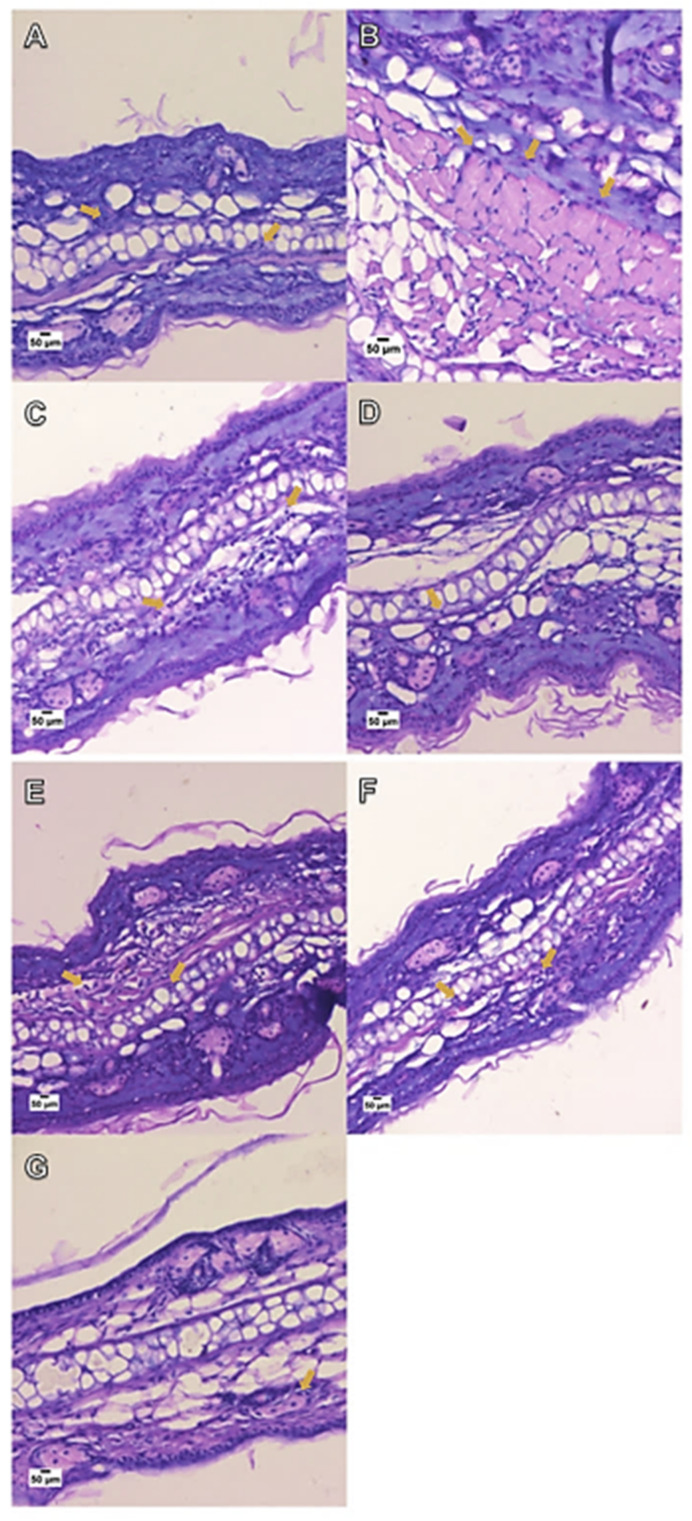
Effect of the hydrogels containing free or nanoencapsulated sesamol topically applied (15 mg/ear) on the polymorphonuclear cell infiltration in the croton-oil-induced ear edema model in *Swiss* mice. The image presents the histological changes (**A**–**G**; hematoxylin–eosin 20× objectives) of the ear tissue of mice at 6 h after the croton oil application or croton oil plus treatments. (**A**) naive; (**B**) croton oil (1 mg/ear) (no treatment); (**C**) croton oil (1 mg/ear) + Hydrogel vehicle; (**D**) croton oil (1 mg/ear) + Hydrogel NC placebo; (**E**) croton oil (1 mg/ear) + Hydrogel free SES; (**F**) croton oil (1 mg/ear) + Hydrogel NC SES; (**G**) croton oil (1 mg/ear) + 0.5% dexamethasone acetate. The arrows indicate the presence of inflammatory cells in the ear tissue. Scale bar = 50 µm.

**Figure 12:**
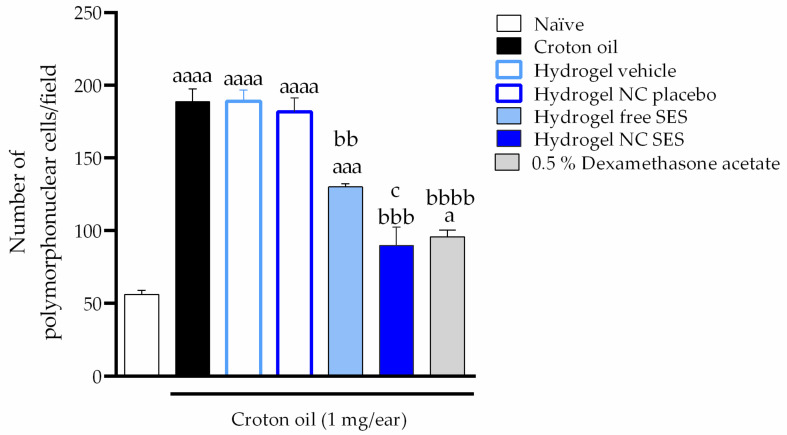
Effect of the hydrogels containing free or nanoencapsulated sesamol topically applied (15 mg/ear) on the polymorphonuclear cell infiltration in the croton-oil-induced ear edema in *Swiss* mice. Topical treatments were applied in mice ear immediately after the croton oil. The samples for the histological technique were collected 6 h after croton oil or croton oil plus treatments. Each bar represents the mean ± SEM (*n* = 3); ^aaaa^ *p* < 0.0001, ^aaa^ *p* < 0.001 and ^a^ *p* < 0.05 show a significant difference when compared to the naïve group; ^bbbb^ *p* < 0.0001, ^bbb^ *p* < 0.001 and ^bb^ *p* < 0.01 show a significant difference when compared to the croton oil control group; ^c^ *p* < 0.05 shows a significant difference when compared to the Hydrogel free SES group. One-way ANOVA followed by post hoc Tukey’s test.

The croton oil application caused polymorphonuclear cell infiltration (Figure 11) in the mice’s ear tissue (189 ± 9 cells per field) when compared to the naïve group (56 ± 3 cells per field). The hydrogels containing the free or nanoencapsulated sesamol inhibited the croton-oil-induced polymorphonuclear cell infiltration with inhibitions of 44.17 ± 1.02% and 74.60 ± 6.27%, respectively. The positive control, 0.5% dexamethasone acetate, caused an inhibition of 70.08 ± 1.29% (Figure 12).

#### 3.9.3. Oxidative Stress Evaluation

**Figure 13:**
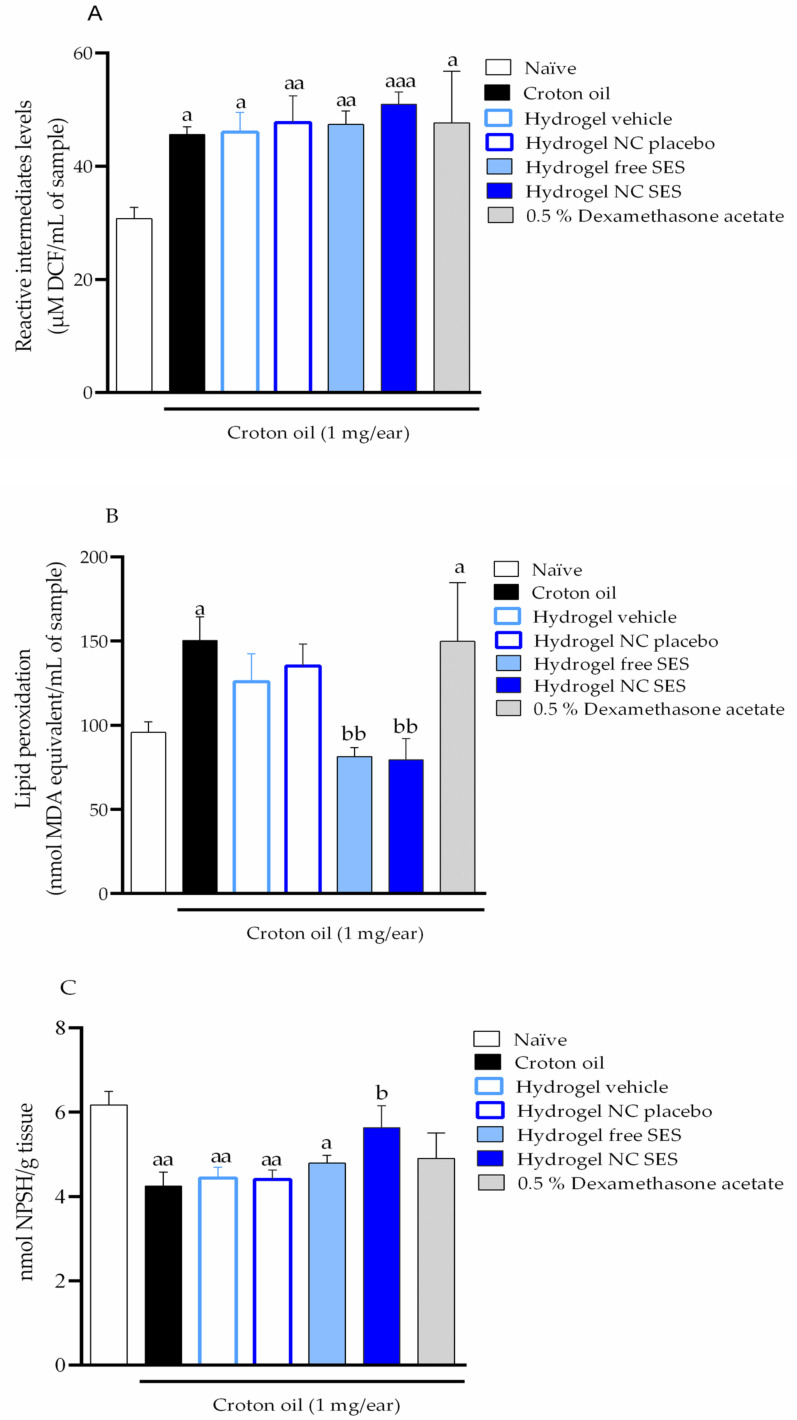
Effect of the hydrogels containing free or nanoencapsulated sesamol topically applied (15 mg/ear) in the mice’s ear on the ROS (**A**), lipid peroxidation (**B**) and nonprotein thiols levels (**C**) in the croton-oil-induced ear edema model in *Swiss* mice. The topical treatments were applied in the mice’s ear immediately after the croton oil. The ear tissue for the assay was collected 6 h after the croton oil or croton oil plus treatments. Each bar represents the mean ± SEM (*n* = 7); ^aaa^ *p* < 0.001, ^aa^ *p* < 0.01 and ^a^ *p* < 0.05 show a significant difference when compared to the naive group; ^b^ *p* < 0.05 and ^bb^ *p* < 0.01 indicate a significant difference when compared to the croton oil control group; one-way ANOVA followed by post hoc Tukey’s test.

The oxidative stress was also assessed through the ROS, TBARS and NPSH levels (Figure 13A–C, respectively). The topical application of the croton oil in the mice’s ear induced an increase of 1.48- and 1.57-fold in the ROS and TBARS levels, respectively. In contrast, the croton oil application reduced the NPSH levels 0.31-fold when compared to the naive group. None of the topical treatments of the hydrogels effectively inhibited the increase in the ROS production in the mice’s ear tissue after the croton oil application. On the other hand, the Hydrogel vehicle and Hydrogel NC placebo inhibited lipid peroxidation by 44.71 ± 30.44% and 27.50 ± 23.62%, respectively, while the Hydrogel free SES and Hydrogel NC SES completely inhibited the lipid peroxidation with an inhibition of 100%. The topical treatment with the Hydrogel free SES and 0.5% dexamethasone acetate restored the NPSH depletion by 28.47 ± 9.66% and 34.17 ± 11.84%, respectively. Importantly, the Hydrogel NC SES restored the NPSH depletion by 71.98 ± 26.98%.

## 4. Discussion

The limitations concerning the effective and safe treatment of irritant contact dermatitis reported in previous studies guided the design of this study [32,33]. In the present work, a nano-based hydrogel containing sesamol presenting anti-inflammatory and antioxidant properties was obtained by the preparation of sesamol-loaded nanocapsule suspension using guar gum as a thickening agent. The results demonstrated that the sesamol nanoencapsulation maximized its pharmacological properties and caused no in vivo toxicity.

Polymeric nanocapsules are promising carriers of drugs due to the fact of their advantages over the conventional pharmaceutical dosage form. These nanoparticles could modify the drug release profile, favoring a prolonged local effect modulating the release to the underlying skin layers, resulting in low systemic absorption [34]. Among the polymers used for the preparation of nanocapsules, ethylcellulose, a cellulose derivative with ethoxyl groups replacing hydroxyls, has been exploited due to the fact of its controlled release properties [16,35]. This way, this polymer was selected to compose the sesamol nanocapsules in order to prolong the drug release.

After the preparation, a physicochemical characterization was performed to elucidate the properties of the nanocapsule suspensions and stability profile. In this context, the sesamol-loaded nanocapsule suspensions had a homogenous distribution in the nanometric range. The zeta potential parameter reflected the surface charge of the nanoparticles and, as expected, the nanocapsule suspensions produced with ethylcellulose polymer exhibited negative values [36]. The sesamol-loaded nanocapsule suspensions showed an encapsulation efficiency of 65%, which was expected due to the low partition coefficient (Log P of 1.29) favoring the sesamol partition to the external aqueous phase [37]. Our results agree with previous studies performed with ethylcellulose nanocapsules presenting a similar qualitative and quantitative composition [16,35,38]. In those studies, the formulations showed the absence of microparticles, an average size of approximately 200 nm, PDI values lower than 0.2, a negative zeta potential and a controlled drug release profile.

Regarding the in vitro release profiles of the sesamol-loaded nanocapsule suspension, it was observed that the nanocapsules provided a sesamol burst effect followed by a prolonged release for 5 h. The advantage of a biphasic release profile is the fast drug onset action followed by a prolonged release to maintain the pharmacological action for longer [39]. The burst effect observed may be due to the sesamol partial nanoencapsulation.

To elucidate the compound stability, the sesamol photodegradation profile was evaluated through its exposure to UVC radiation. The results obtained in the photostability evaluation demonstrated that the sesamol-loaded nanocapsule suspension attenuated the sesamol photodegradation compared to its free form, which reinforces the adequate photoprotection conferred by the nanocapsules. This result could be attributed to the nanometric size that allows it to scatter and reflect the UVC radiation [40]. In addition, our results showed that the nanoencapsulation increased the radical scavenging activity of DPPH and ABTS radicals in comparison to the free sesamol. In this sense, previous studies suggested that the nanometric size could provide a superior contact surface area between the H donator and pro-oxidant molecules, facilitating the access of the hydrogen atom to the radical [41].

Hydrogels containing the sesamol-loaded nanocapsule suspension were proposed, because the nanocapsule formulations were obtained in liquid form. Therefore, a gelling agent was necessary to achieve suitable properties for cutaneous application. Guar gum, a biocompatible and nontoxic microbial polysaccharide, was applied as the thickening agent [14,42]. The physicochemical characterization of the developed hydrogel showed that the nanocapsule parameters in terms of the average size, polydispersity index (PDI) and pH were not significantly affected in comparison with those obtained for the nanocapsules in the aqueous suspension. This way, the nanocapsules kept their characteristics, even after being submitted to the thickness process to form the hydrogel.

Easy spreadability and suitable flow characteristics are essential properties of pharmaceutical dosage forms intended for cutaneous application [43]. In this context, both hydrogels exhibited pseudoplastic flow indicating that the viscosity decreased as the applied shear stress increased, which was mathematically described by the Ostwald-de-Waele model, demonstrating that the hydrogels spread under a low shear stress. This behavior is desirable, because it increases the residence time and contact with the injured tissue.

The ban on the use of animal models within the cosmetics industry has led to an urgent need for alternative test methods to assess new compounds. The HET-CAM alternative method was validated for eye irritation tests because of its high sensitivity [44]. However, it can be applied to prevent irritation in other mucous membranes, such as the skin [40,45]. In this study, the free or nanoencapsulated sesamol forms did not show any irritant effect, suggesting that the developed formulations are safe for the cutaneous application.

Inflammation models induced by irritant agents in rodents are a preclinical approach for investigating new therapeutic compounds with topical anti-inflammatory effects. In the present study, an in vivo model of the croton-oil-induced irritant contact dermatitis was performed to assess the therapeutic potential of the hydrogel containing sesamol in the free or nanoencapsulated form. Croton oil, because of its main constituent, 12-O-tetradecanoylphorbol-13-acetate (TPA), is recognized as a compound able to experimentally induce irritant contact dermatitis [46]. In the present study, the croton oil application induced skin inflammation characterized by edema formation and leukocyte migration to the injured tissue. All of these inflammatory damages were mitigated by the cutaneous application of the Hydrogel NC SES but not by the hydrogel containing the free sesamol.

According to these results, we may raise the hypothesis that there is a fast sesamol permeation decreasing its residence time in the skin layers limiting the anti-inflammatory effect. Corroborating this, the in vitro hydrogels’ release profile demonstrated that the sesamol-containing hydrogel in the free form presented a fast compound diffusion through the dialysis cellulose membrane. The ex vivo skin permeation profile reinforced favorable the sesamol physicochemical properties facilitating its fast permeation. Therefore, it may be said that the sesamol’s topical application probably reached systemic circulation, as it was shown by the high concentration in the receptor medium compartment and by the low retention in the superficial skin layers.

On the other hand, the sesamol-containing hydrogels in the nanoencapsulated form showed a prolonged release profile in comparison to the hydrogel containing free sesamol. The ex vivo permeation results indicate that the Hydrogel NC SES provided a lower sesamol concentration in the dermis and receptor medium compartments when compared to the Hydrogel free SES. These results are in agreement with the characteristic of the polymer used in the sesamol-loaded nanocapsule preparation, because ethylcellulose is a water-insoluble polymer used to promote prolonged-release profiles.

Irritant contact dermatitis is associated with the local release of inflammatory mediators such as reactive oxygen species, and it is regulated by the antioxidant defense mechanism [47]. Oxidative stress is defined as a redox status imbalance of pro-oxidant and antioxidant systems [48]. The oxidative stress status is developed in injured tissue because inflammatory cells, such as neutrophils and macrophages, produce large amounts of ROS [49]. Redox imbalance is implicated in the structural damage of cells’ macromolecules, for instance, lipids and proteins. In this context, the TBARS content was used, such as a lipid damage marker [50]. Moreover, NPSH is an important nonenzymatic antioxidant that scavenges reactive oxygen metabolites, such as superoxide (O^2−^) and hydroxyl (OH^−^) ions, in the biological system [51].

Our present results indicate the croton oil application induced oxidative stress, characterized by increased levels of ROS and TBARS, while the NPSH levels were reduced. The cutaneous application of the hydrogels containing sesamol in the free or nanoencapsulated form significantly lowered the TBARS levels. However, only the cutaneous treatment with the Hydrogel NC SES reversed the croton-oil-induced decreased NPSH levels. This effect was not observed in the group treated with the free sesamol hydrogel, and this could be attributed to the fast permeation through the skin’s layers, but it was not effective concerning the NPSH levels modulation. Therefore, these findings reinforce the sesamol antioxidant property and the nanoencapsulation benefits because it modulates the oxidative redox homeostasis and reduces the croton-oil-induced acute inflammation damage.

## 5. Conclusions

The nanocapsule suspensions presented adequate physicochemical characteristics and were able to enhance the sesamol’s photostability and radical scavenger properties. The hydrogels had adequate physicochemical characteristics and appropriate behavior for cutaneous application. In addition, the hydrogel containing sesamol in the nanoencapsulated form showed a prolonged release profile, which supports the increase in its residence time in the skin’s layers, and an improvement in the pharmacological action in a preclinical model of irritant contact dermatitis. The findings of the in vivo study indicate that the hydrogel containing sesamol in the nanoencapsulated form modulated the oxidative stress and demonstrated an anti-inflammatory efficacy similar to dexamethasone acetate, indicating that it could be a promissory therapy to treat inflammatory skin diseases.

## Figures and Tables

**Table 1:** Characterization parameters of the sesamol-loaded nanocapsule suspension (NC SES) and its respective unloaded (NC placebo) formulation.

Parameter	NC SES	NC Placebo
D[4;3] (µm)	0.135 ± 0.008	0.137 ± 0.024
SPAN	0.564 ± 0.111	0.557 ± 0.286
Average diameter (nm)	127 ± 2	131 ± 2
PDI ^a^	0.11 ± 0.02	0.09 ± 0.02
ZP ^b^ (mV)	−20.4 ± 0.4	−18.6 ± 1.4
pH	5.2 ± 0.0	5.0 ± 0.0
Sesamol content (%)	100.1 ± 0.8	- ^c^
	After 15 days	
D[4;3] (µm)	0.137 ± 0.008	0.133 ± 0.004
SPAN	0.514 ± 0.060	0.570 ± 0.110
Average diameter (nm)	134 ± 1	135 ± 3
PDI ^a^	0.12 ± 0.01	0.11 ± 0.01
ZP ^b^ (mV)	−22.7 ± 1.2	−22.1 ± 0.9
pH	5.4 ± 0.0	5.1 ± 0.0
Sesamol content (%)	96.1 ± 1.1	- ^c^
	After 30 days	
D[4;3] (µm)	0.770 * ± 1.040	0.430 * ± 0.310
SPAN	0.850 * ± 0.150	1.050 * ± 0.650
Average diameter (nm)	131 ± 1	133 ± 3
PDI	0.12 ± 0.01	0.12 ± 0.00
ZP (mV)	−21.1 ± 0.0	−19.7 ± 1.7
pH	5.7 ± 0.1	5.4 ± 0.2
Sesamol content (%)	101.2 ± 1.5	- ^c^

^a^ PDI: polydispersity index. ^b^ PZ: zeta potential. ^c^ -: Not applicable. The values are reported as the mean ± SD of three batches/formulation. All data were analyzed using the unpaired Student’s *t*-test (individual times) and one-way ANOVA of repeated measures followed by post hoc Tukey’s test (stability evaluations). The asterisks (*) denote a significant difference in comparison to the initial time (*p* < 0.05).

**Table 2:** Characterization parameters of the hydrogels.

	AD ^a^ (nm)	PDI ^b^	pH	SES Content (%)
Hydrogel NC SES	127 ± 3	0.11 ± 0.02	6.5 ± 0.0	99.6 ± 1.6
Hydrogel NC placebo	126 ± 5	0.12 ± 0.03	6.4 ± 0.3	- ^c^
Hydrogel free SES	- ^c^	- ^c^	6.3 ± 0.1	99.7 ± 0.6
Hydrogel vehicle	403 ± 3	0.62 ± 2.6	6.4 ± 0.2	- ^c^

^a^ AD: average diameter. ^b^ PDI: polydispersity index. ^c^ -: not applicable. The values are reported as the mean ± SD of three batches/formulations. The data were analyzed using one-way ANOVA (*p* > 0.05).

**Table 3:** Regression coefficients (r^2^) and mathematical equations to models of Bingham, Casson, and Ostwald-de-Waele.

	Mathematical Models (regression coefficients (r^2^))		
Formulation	Bingham	Casson	Ostwald-de-Waele
Hydrogel NC SES	0.749 ± 0.015	0.856 ± 0.008	0.945 ± 0.004
Hydrogel NC placebo	0.657 ± 0.168	0.753 ± 0.180	0.949 ± 0.010
Hydrogel free SES	0.823 ± 0.005	0.923 ± 0.005	0.987 ± 0.003
Hydrogel vehicle	0.775 ± 0.029	0.878 ± 0.024	0.957 ± 0.015
	Ostwald-de-Waele		
Formulation	ƞ ^a^		k ^b^
Hydrogel NC SES	0.190 ± 0.031		17,412 ± 191
Hydrogel NC placebo	0.230 ± 0.050		17,834 ± 480
Hydrogel free SES	0.130 ± 0.001		17,932 ± 671
Hydrogel vehicle	0.166 ± 0.020		19,873 ± 328

^a^ ƞ: Flow index. ^b^ k: Consistency index. The experiment was performed in triplicate for each hydrogel, and the values are reported as the mean ± SD. All data were analyzed by ordinary one-way ANOVA (*p* > 0.05).

## Data Availability

Not applicable.

## Supplementary Materials

The following supporting information can be downloaded at: https://www.mdpi.com/article/10.3390/pharmaceutics15010285/s1, Figure S1. Chemical structure of guar gum; Figure S2: Representative chromatographic profiles of sesamol (15 µg/mL - chromatogram A) and NC placebo (chromatogram B) at 297 nm; Figure S3: Representative granulometric profiles determined by laser diffraction at their initial time of NC SES (Figure 3SA) and NC placebo (Figure 3SB), respectively. The nanoparticles population is in the nanometric range (<1 µm); Figure S4: Representative images of size distribution curves determined by dynamic light scattering (DLS) at their initial time of the NC SES (A) and NC placebo (B), respectively.
