# Supplementary material for: Development of Guar Gum Hydrogel Containing Sesamol-Loaded Nanocapsules Designed for Irritant Contact Dermatitis Treatment Induced by Croton Oil Application
Source: Pharmaceutics. 2023 Jan 14;15(1):285. doi: 10.3390/pharmaceutics15010285 (PMC9861215; doi:10.3390/pharmaceutics15010285)
Supplement: Supplementary file 1 [file pharmaceutics-15-00285-s001.zip › pharmaceutics-2147899-supplementary.pdf]

pharmaceutics

Supplementary Materials: Development of guar gum hydrogel containing sesamol-loaded nanocapsules designed for the irritant contact dermatitis treatment induced by the croton oil application

Vinicius Costa Prado; Kauani Moenke; Bárbara Felin Osmari; Natháli Schopf Pegoraro; Sara Marchesan de Oliveira; Letícia Cruz

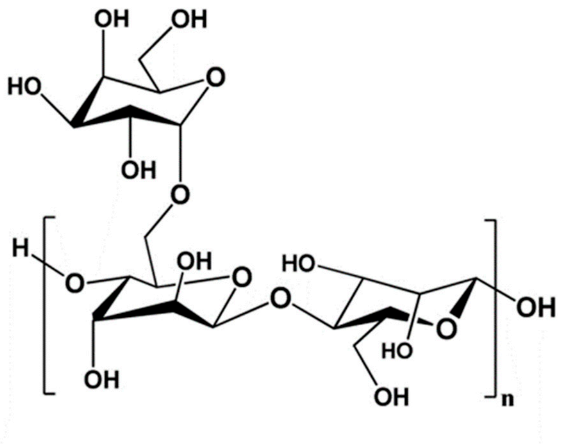

Figure S1. Chemical structure of guar gum.

A

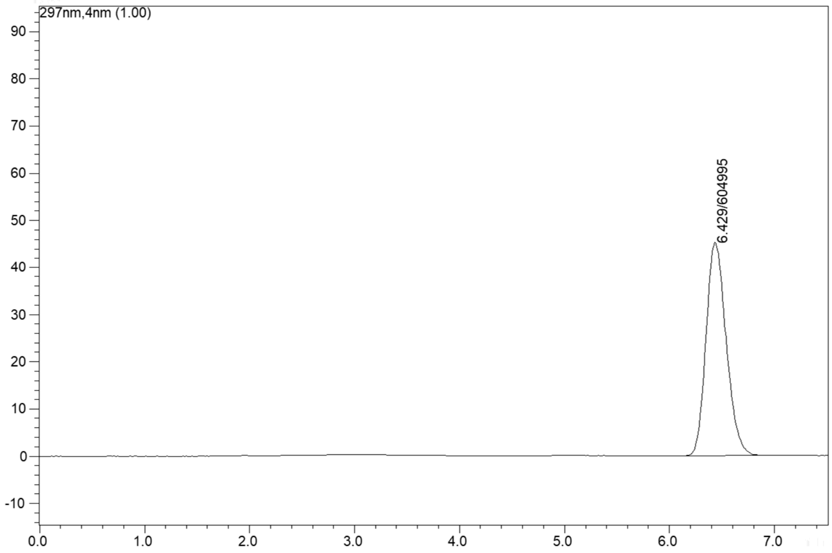

B

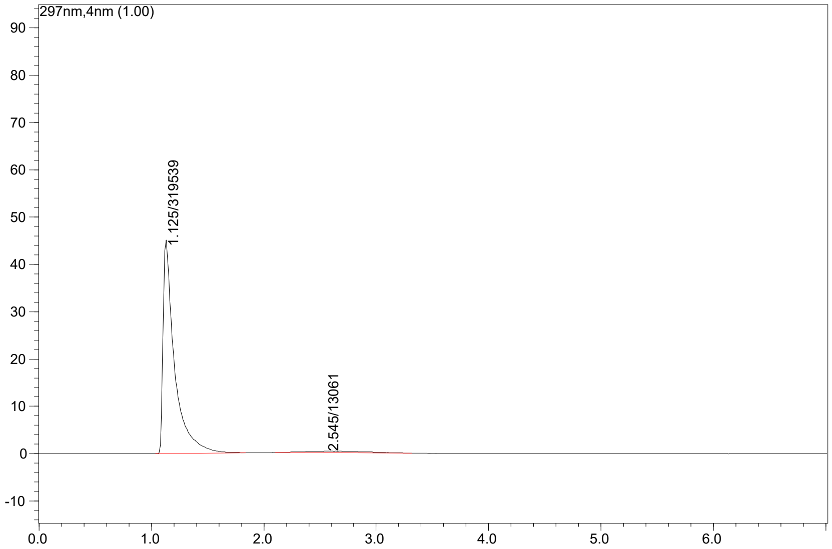

Figure S2. Representative chromatographic profiles of sesamol (15 µg/mL - chromatogram A) and NC placebo (chromatogram B) at 297 nm.

A

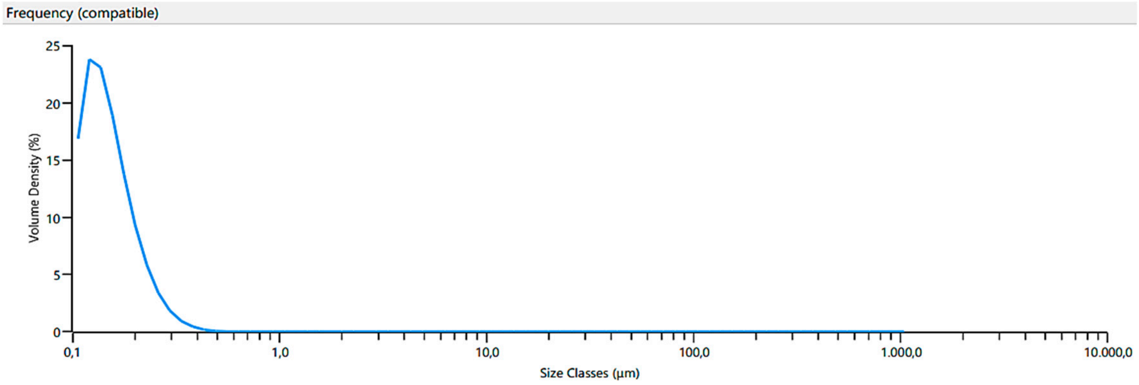

B

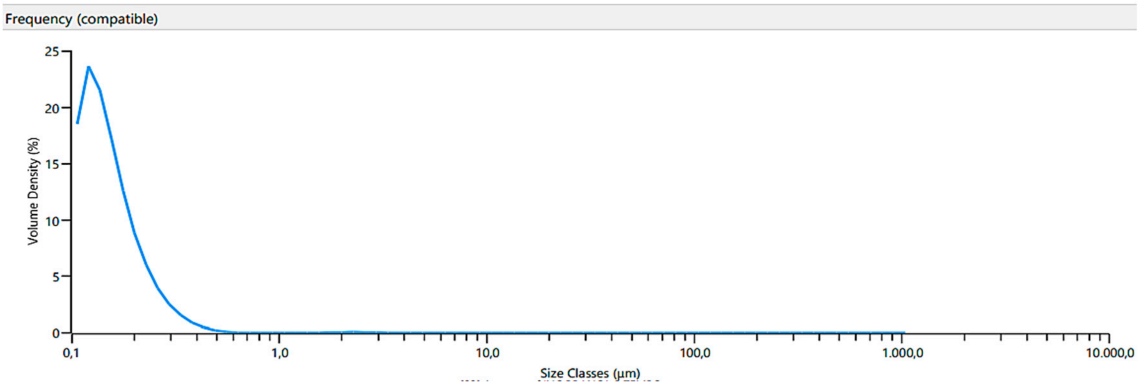

Figure S3. Representative granulometric profiles determined by laser diffraction at their initial time of NC SES (Figure 3SA) and NC placebo (Figure 3SB), respectively. The nanoparticles population is in the nanometric range (< 1 µm).

A

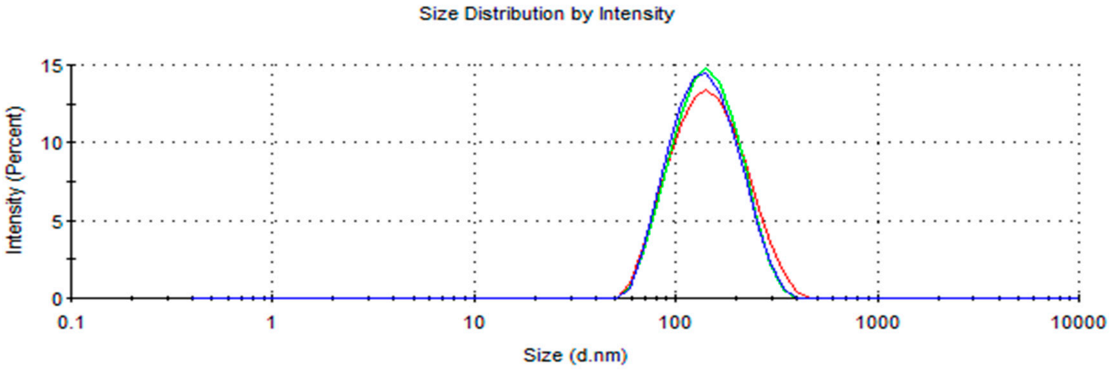

B

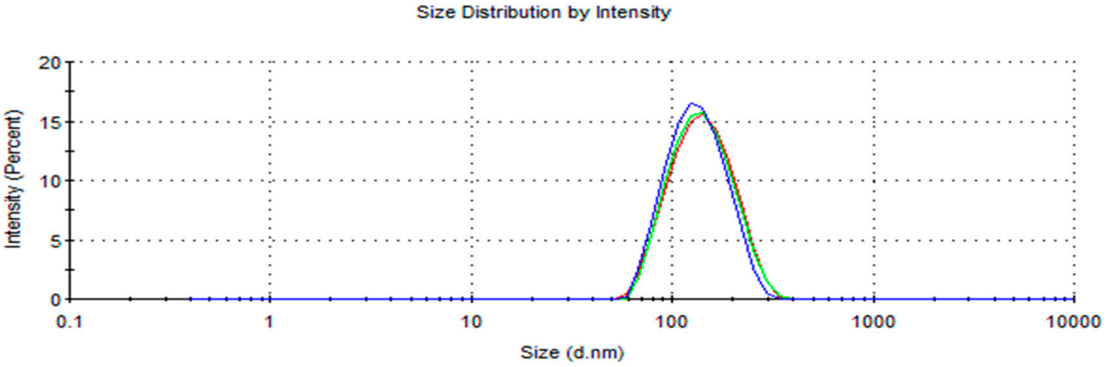

Figure S4. Representative images of size distribution curves determined by dynamic light scattering (DLS) at their initial time of the NC SES (A) and NC placebo (B), respectively.
